# Choroidal Melanoma: A Case Series From Malaysia

**DOI:** 10.7759/cureus.31105

**Published:** 2022-11-04

**Authors:** Chia Yaw Teoh, Wan Mariny W Md Kasim, Talib Norlaila

**Affiliations:** 1 Ophthalmology, Hospital Serdang, Selangor, MYS; 2 Ophthalmology, Hospital Serdang, Kajang, MYS

**Keywords:** case series, uveal melanoma, uveal, malaysia, melanoma, choroidal melanoma

## Abstract

Introduction: Choroidal melanoma is one of the subtypes of uveal melanoma and a relatively rare ophthalmic malignancy worldwide. However, it is scarce in Asian countries like Malaysia.

Purpose: We present eight cases of patients with choroidal melanoma referred to Hospital Serdang, a tertiary centre for oculoplastic issues, from 2021 to 2022.

Method: A retrospective case series of patients diagnosed with choroidal melanoma and referred to the Oculoplastic Clinic, Department of Ophthalmology, Hospital Serdang, was undertaken.

Results: A total of eight cases of choroidal melanoma were identified, with a median age of 65 years. Six of them were female, and two were male. There were five of Malay ethnicity, and three were Chinese. Progressive reduced vision (n = 6), noticeable ocular mass (n = 3), and visual field defect (n = 2) were the most common presenting symptoms. The mean tumour thickness was 21.25 mm (range = 10-56 mm), and the largest basal diameter ranged from 10 mm to 53 mm (mean = 22.5 mm). Most were large tumours (n = 7) and above the T3a stage. The level of lactate dehydrogenase (LDH) was elevated in three cases. Histopathologically, five of the tumours were epithelioid, while the other three were mixed types. All patients underwent enucleation (n = 5) and exenteration (n = 3) with one recurrence. One patient had liver metastasis on the diagnosis of choroidal melanoma.

Conclusion: In Malaysia, there is an increasing number of cases of choroidal melanoma. Clinical evaluation remained the mainstay of diagnosis. Treatment should be based on multifactorial prognostication in addition to tumour size. The LDH level may be necessary for providing inexpensive but valuable prognostication and monitoring markers.

## Introduction

Uveal melanoma is a relatively rare ophthalmic malignancy and the rarest form of melanoma worldwide, with an annual rate of five to six cases per million in the United States of America, Australia, and Europe [1]. On the contrary, it has been noted that Asia-Pacific countries have a much lower incidence of uveal melanoma as compared to the world, ranging from 0.25 to 0.64 cases per million annually in 2021 [1]. The iris colour could partly explain the rarity of tumours among Asians; lighter iris carries more pheomelanin and is associated with increased genotoxic damage, accumulating over time, while darker iris has more eumelanin than pheomelanin, hence the lesser risk of developing uveal melanoma [2].

In Malaysia, the incidence of choroidal melanoma was lower than in Asian countries, with only eight cases reported from 2012 to 2016. The last reported choroidal melanoma was in 2020, as a case report [3,4]. Singapore, a country with a similar culture and ethnic background to Malaysia, has provided national data regarding ophthalmic malignancies from 1996 to 2016, revealing 21 out of 297 patients had malignant uveal melanoma, whereby choroidal melanoma comprised 16 of them in 20 years, signifying the scarcity of choroidal melanoma among the Asian population [5]. Thus, this case series would like to provide valuable information regarding the latest cases of choroidal melanoma encountered by tertiary referral centres in Malaysia.

## Materials and methods

A retrospective case series of patients diagnosed with choroidal melanoma and referred to the Oculoplastic Clinic, Department of Ophthalmology, Hospital Serdang, from 2021 to 2022, was undertaken. Patient demographic data, clinical presentation, blood investigations, tumour characteristics, treatment, outcomes, and complications were tabulated and discussed.

The diagnosis of choroidal melanoma was made through clinical ophthalmic evaluation with the aid of ophthalmic ultrasound and computed tomography of the orbit, with further expert evaluation from an experienced oculoplastic subspecialist. The diagnosis was supported by lactate dehydrogenase (LDH) level for prognostication and formal histopathological examination by experienced pathologists.

## Results

Eight cases of choroidal melanoma were identified and tabulated. In eight patients, females (6, 75%) outnumbered males (2, 25%). The median age of patients was 65 years. Regarding ethnicity, five (62.5%) of them were Malay, and the others were of Chinese race (3, 37.5%). Out of eight patients, three (37.5%) had another ocular naevus present aside from choroidal melanoma, with two (25%) over the eyelid and one on the iris. The summary is depicted in Table 1.

**Table 1:** Demographic information of patients

Patient No.	Age	Gender	Ethnic	Occupation	Family history of cancer	Smoking history	Ocular naevus	Comorbidity
1	56	Female	Malay	Housewife	No	No	Eyelid	Nil
2	66	Female	Malay	Housewife	No	No	Eyelid	Nil
3	71	Female	Chinese	Cleaner	No	No	Absent	Metabolic syndrome
4	64	Male	Chinese	Metal factory labourer	No	No	Absent	Nil
5	73	Female	Malay	Housewife	No	No	Absent	Hypertension
6	23	Female	Malay	Housewife	No	Yes	Iris	Nil
7	67	Female	Malay	Housewife	Yes	No	Absent	Nil
8	50	Male	Chinese	Lorry driver	Yes	No	Absent	Nil

**Figure 1:**
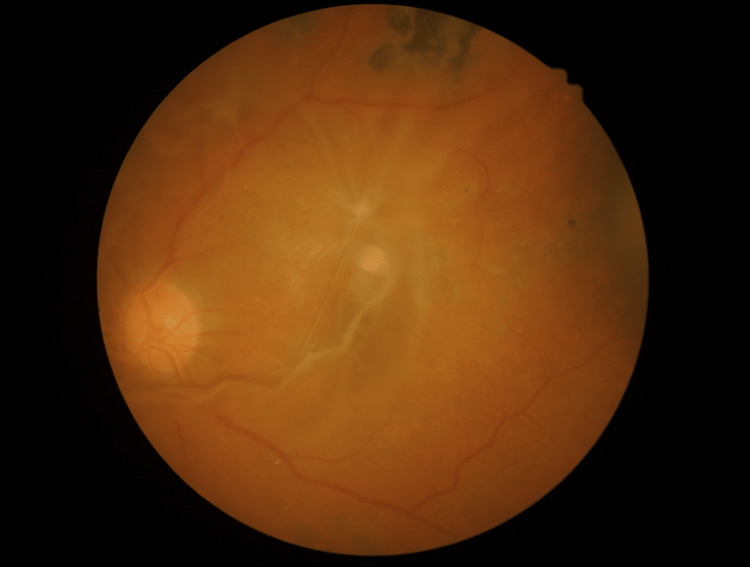
A fundus photo of the left eye showed vitreous condensation over the macula with traction on the supertemporal vascular arcade and amelanotic choroidal lesion temporal to the macula in the lasered eye in the patient treated for proliferative diabetic retinopathy

According to Table 2, the most common clinical presentation was a progressive reduced vision (n = 6, 75%), followed by noticeable ocular mass (n = 3, 37.5%), visual field defect (n = 2, 25%), increasing floaters (n = 1, 12.5%), and constitutional symptoms (n = 1, 12.5%). In addition, two patients had first-degree relatives with a family history of malignancy. Still, none of them had a family history of ocular malignancy, namely, nasopharyngeal carcinoma and breast cancer. One of the patients was a second-hand smoker. One patient worked in a metal processing factory, but none had an occupation with prolonged sunlight exposure. One of the patients had been treated for proliferative diabetic retinopathy with unresolved vitreous haemorrhage refractory to pan-retinal photocoagulation, as per Figure 1. One had neovascular glaucoma during the presentation, one had a ciliary body melanoma, and another had a history of strabismus surgery during childhood. Further blood investigations showed negative tumour markers but elevated LDH levels in three patients.

**Table 2:** Clinical presentation of patients ^1 ^Represents the presented visual acuity of the affected eye. HM: hand movement; NPL: no perception of light; PH: vision after pinhole.

Patient No.	Presenting	Symptoms	Other ocular issues	Duration of presentation	Lactate dehydrogenase
	Visual acuity^1^				
1	HM	Decreased vision, floaters	Nil	7 months	695
2	NPL	Decreased vision, mass	Neovascular glaucoma	4 months	-
3	CF	Decreased vision	Unresolved vitreous haemorrhage	3 months	372
4	NPL	Decreased vision, mass	Childhood strabismus surgery	7 years	-
5	Unaided 6/36	Vision field defect	Nil	3 weeks	-
	PH 6/24				
6	Unaided 6/60	Visual field defect	Exudative retinal detachment, ciliary body melanoma	2 weeks	-
	PH 6/24				
7	NPL	Decreased vision, constitutional symptoms	Nil	1 month	202
8	NPL	Decreased vision, mass	Nil	1 year	1301

Table 3 illustrates the tumour characteristics. The tumour was tabulated by the features of choroidal melanoma described by Shields et al. using the mnemonic "To find small ocular melanoma using helpful hints daily," which stands for thickness, fluid, symptoms, overlying orange pigmentation, margin from the optic disc, ultrasonographic hollowness, halo absence, and drusen absence [6]. The tumour size was classified using the Collaborative Ocular Melanoma Study (COMS) and the tumour staging described by the American Joint Committee on Cancer (AJCC) 8th Edition. On average, the tumour's thickness was 21.25 mm, ranging from 10 mm to 56 mm, while the largest basal diameter was, on average, 22.5 mm, which ran from 10 mm to 53 mm. With these eight predictive features, most of the tumours had four features (n = 5, 62.5%) and three features (n = 2, 25%). All the tumours were graded T3a and above, with primarily large tumours (n = 7, 87.5%). Most of the tumours were epithelioid type (n = 5, 62.5%), while others were mixed type (n = 3, 37.5%), as tabulated in Table 4.

**Table 3:** Tumour characteristics DD: disc diameter.

Patient No.	Largest basal diameter (mm)	Tumour thickness (mm)	Sub-retinal fluid	Symptoms	Orange pigment	Margin from the optic disc	Ultrasonographic hollowness	Halo	Drusen
1	12	10	No	Yes	Yes	4DD	No	No	No
2	15	20	No	Yes	No	Involved	Yes	No	No
3	10	10	No	Yes	No	3DD	Yes	No	No
4	21	20	No	Yes	No	Involved	Yes	No	No
5	40	28	No	Yes	No	2DD	No	No	No
6	14	13	No	Yes	No	4DD	No	No	No
7	15	13	No	Yes	No	4DD	Yes	No	No
8	53	56	No	Yes	Yes	Involved	No	No	No

**Table 4:** Treatment, outcome, and complication ^1^ Tumour grade classification using American Joint Committee on Cancer, 8th Edition [7]. ^2 ^Tumour size classification using the Collaborative Ocular Melanoma Study.

Patient No.	Treatment	Histopathological examination	Tumour grade^1^ (size)^2^	Complication	Duration of follow-up (month)	Current status	Metastasis
1	Enucleation	Mixed	T3aN0M0 (large)	Nil	12	Well	None
2	Exenteration	Mixed	T4eN0M0 (large)	Recurrence	14	Well	None
3	Enucleation	Epithelioid	T3aN0M0 (medium)	Nil	10	Well	None
4	Enucleation	Epithelioid	T4bN0M0 (large)	Nil	10	Well	Liver
5	Exenteration	Epithelioid	T4eN0M1 (large)	Nil	8	Well	None
6	Enucleation	Epithelioid	T3bN0M0 (large)	Nil	5	Well	None
7	Enucleation	Epithelioid	T3aN0M0 (large)	Nil	4	Well	None
8	Exenteration	Mixed	T4eN0M0 (large)	Nil	3	Well	None

All patients were subjected to the surgical interventions described in Table 4. Five of them underwent enucleation, while three of them had exenteration. None of them had immediate surgical complications. However, one of them had a tumour recurrence after exenteration, while another patient was found to have liver metastasis before enucleation. All of them were striving well over a mean duration of 8.25 months. Given the nature of surgical intervention in this study, the final visual acuity for the affected eye is not applicable.

## Discussion

Uveal melanoma is a malignancy of melanocytes found in the eye globe's uveal tract (iris, ciliary body, and choroid). Choroidal melanoma is considered the most common among all subtypes of uveal melanoma despite its rarity among the Asian population.

In this case series, there was an increased number of cases of choroidal melanoma in Malaysia in two years. There was a preference of patients towards females with a median age of 65 years old, which was consistent with the previous Malaysian case series published in 2017. A worldwide review showed predominantly males with a younger age group [1,3]. Additionally, ocular naevus was found in nearly one-third of patients, which was a risk factor for developing uveal melanoma. One patient worked as a metal factory labourer, which might increase the risk of uveal melanoma. Another study showed a similar list of risk factors for developing uveal melanoma, such as Caucasian, oculodermal melanocytosis, light irises, sunlight, and occupation (arc welders and airline workers), which correspond to our patients’ demographic findings [8]. Kaliki et al. also provided additional risk factors and information about uveal melanoma. In addition to the previously mentioned risk factors, fairer skin colour and ease of skin tanning were significant risk factors for uveal melanoma. At the same time, sunlight exposure has less concrete evidence to contribute to uveal melanoma [9]. The clinical presentation of uveal melanoma in this case series was decreased vision, noticeable ocular mass, and visual field defect, which was nearly similar to other epidemiological studies mentioned [1,3,5].

Most patients with a high clinical score suggest an excellent screening tool for identifying choroidal melanoma [6]. There was a larger mean basal diameter of the tumour, which was consistent with the study, yet most were large tumours compared to the survey with many medium tumours [1]. This could be due to delayed hospital access from the coronavirus disease 2019 (COVID-19) pandemic. In addition, tumour markers like alpha-fetoprotein (AFP), Ca125, CA19-9, and carcinoembryonic antigen (CEA) were negative for all patients with elevated LDH in three of them, the highest corresponding to the highest tumour grading using AJCC tumour criteria. Unfortunately, not all the patients had LDH taken, which could have contributed to a better picture for using LDH level as prognostication and aid in diagnosing uveal melanoma. Nonetheless, the research studies the tumour markers in uveal melanoma in terms of diagnosis and prognostication. Bande Rodriguez and colleagues have provided perspectives regarding blood biomarkers in uveal melanoma. In their review, carcinoembryonic antigen (CEACAM-1) showed an increased level in uveal melanoma but was rarely used in uveal melanoma as it may be confused with other malignancies [10]. Also, the liver enzyme was reviewed and found to be of limited use due to its low sensitivity, whereas LDH had the highest sensitivity of 67% [10]. However, LDH level has been proven helpful in various studies, including uveal melanoma, due to increased glycolysis rate in tumour cells, resulting in increased LDH activity [11]. In a recent meta-analysis, LDH level helps determine overall survival and time to hepatic progression before treatment initiation, as well as a potential biomarker in prognostication of anti-tumour therapy [12,13].

In this study, there was a high proportion of tumours of the epithelioid type as compared to mixed cytology in terms of histopathological view. This finding differed from past studies in Malaysia and Singapore, which comprised mainly spindle cells and mixed type [1,3-5]. Also, one study showed that epithelioid cytology was more commonly present in conjunctival melanoma (66.7%) than in uveal melanoma (48%) [14]. Epithelioid-type choroidal melanoma was an aggressive type associated with poor clinical outcomes [8]. Kaliki et al. have provided a comprehensive list of characteristics ranging from clinical, histological, cytogenetics, and transcriptomic features regarding choroidal melanoma prognostication. These characteristics included being elderly, male, having a sizeable basal diameter and thickness, having advanced AJCC staging, and having epithelioid cytology [9]. In light of this finding, it was understandable that all of the patients in this study did not receive globe-preserving therapy (enucleation and exenteration), not to mention that patient wishes were considered in treatment decisions.

Among eight patients, one had a recurrence, one had pre-treatment liver metastasis, and both were associated with an advanced tumour stage. Tan and colleagues have analysed the recurrence-free survival and overall survival in non-metastatic uveal melanoma that underwent curative treatment. Higher T stage of the tumour, smoking history, and age greater than 40 years were associated with worse survival and higher recurrence, with an overall five-year recurrence-free survival rate of 56.8% and 76.6%, respectively [14]. The International Rare Cancers Initiative (IRCI) study also conducted a meta-analysis for the survival of 912 patients with uveal melanoma. As a result, the median progression-free survival (PFS) was 3.3 months and a PFS rate of 27% in six months, with a median overall survival (OS) of 10.2 months and a one-year OS rate of 43% [15].

In terms of treatment, all patients underwent enucleation and exenteration. Kaliki et al. have discussed the nature, risks, and disease recurrence rate for several treatments for uveal melanoma, including close monitoring, globe-salvaging treatment, enucleation, and exenteration. The study recommended enucleation in large tumours, poor visual potential, moderate extraocular extension, and exenteration in cases of extensive extraocular extension [9]. Compared to the landmark studies, COMS, Honavar has provided invaluable insight into our current understanding of uveal melanoma. While COMS treatment depends on the tumour size alone, the latest studies use multifactorial prognostication to decide treatment options. Specifically, earlier local treatment, even in a small tumour, should be considered, and adjuvant therapy should be thought of when there is a high metastatic risk, noting that an attempt should be made to conserve the eye and vision whenever possible [16]. Thus far, one of the patients had a recurrence within 14 months of follow-up, and one had liver metastasis before enucleation, while all patients are still striving well during follow-up this far.

## Conclusions

This study showed an increasing trend of choroidal melanoma in Malaysia over two years. The demographic characteristics associated with choroidal melanoma were elderly, female, and the presence of ocular naevus. Most patients presented with decreased vision, a noticeable ocular mass, and a visual field defect. Clinical evaluation remains essential in the diagnosis of choroidal melanoma. The majority of patients in this study had a larger basal diameter of tumour, advanced tumour stage, and epithelioid cytology predominates, suggesting poor prognosis. Treatment of choroidal melanoma should be tailored to not only tumour size but also multifactorial prognostication to optimise clinical outcomes between conserving remaining vision potential with risk of recurrence and metastasis. Aside from clinical features, tumour size, and staging, LDH level might help determine the prognosis and treatment for the patient.
